# The Book World of Medicine and Science

**Published:** 1903-07-04

**Authors:** 


### The Book World of Medicine and Science.

Famishing London: A Study of the Unemployed and Unemployable. By F. A. McKenzie. London: Hodder and Stoughton, 27 Paternoster Row. Price 1s.

Mr. McKenzie's little book forms interesting but very depressing reading. The descriptions of poverty as the author saw it in Canning Town we need not touch on. Some portions of the book dealing with this, especially as it affected the children, were printed in the *Daily Mail*, and happily had the effect of raising a good deal of money for the sufferers. But the hungry children, like the processions of unemployed, are only a symptom of the disease which Mr. McKenzie treats of, and the disease is evidently a serious one. As he says: "The unemployed processions may have been a mistake, but they were also a menace and a warning. The winter of 1903 has been one of exceptional mildness. Trade throughout the country has been on the whole good. Our great industries, with the noted exceptions of ship-building, shipping, and cotton, are prosperous. And yet at this time 4,000 men on one afternoon will parade the streets of London and beg, while tens of thousands more, too self-respecting to plead for alms, too shamefaced to make their poverty known, are starving in silence." The exceptions to the general prosperity are so serious that perhaps they ought to get more than a casual mention, but only the last of them—shipping—greatly affects London. And it is to the decline of the shipping trade of London that Mr. McKenzie traces part of the distress that has marked this winter. "The shipping entering the port of London to discharge from foreign ports during December, 1901, came to 566,000 tons. In December, 1902, it was only 518,000. In January, 1902, the figures were 516,000; in January, 1903, only 466,000 tons. These figures mean much more than shortage at the docks. The imports are largely the barometer of trade. Less men are employed at the docks, fewer at warehouses, and fewer in a hundred other ways." The other cause is, in Mr. McKenzie's opinion, the alien immigration. He does not indulge in that rabid abuse of the foreigner which is popular in some quarters at present. But he says:—"The coming of the alien has meant much harder times for our own poor. The very virtues of the Eastern Jews cause this. If they were inferior to our slum inhabitants, there would be little trouble. But they are not. Taking them as a whole, they are the superior people. They are more sober, they are more thrifty, they work harder, they have stronger constitutions, they are banded together by the freemasonry of race and religion, and they supplant our own folk, taking their work from them, driving them from their homes." It is this superiority that, in one sense, causes the trouble. The Government of England—of England, which has for generations past been the refuge of the distressed, is unwilling to refuse admittance to sober, thrifty, industrious people, many of whom are fleeing from intolerable oppression and persecution. If these aliens were all criminals or even anarchists, as some newspapers try to make out, there would be little hesitation in refusing them admittance or deporting them when they had contrived to land on our shores. Moreover, it can hardly be contended that as yet the coming of the alien forms a national danger. It is, as one critic scornfully said, "A Whitechapel question." But that does not make it any the easier for Whitechapel. There are two solutions of the imbroglio. One is to refuse admission to foreigners and even to deport those who have already made good their settlement. This would undoubtedly be popular at the moment. But when the prices of the poorer sorts of clothing and shoes, such as the foreign Jews make, became dearer, those who now cry out the loudest for the expulsion of the alien would be the first to complain. If the worker is to get bigger wages—he being admittedly an inferior worker—the consumer will have to pay. Perhaps higher considerations may make this worth while, but it is well to know the facts in advance. The other is for our own people either to go to the rural districts of England, where labour cannot at present be got, or to emigrate to one of the colonies. Sentiment is against this permitting foreigners to squeeze the native population away, but if we put sentiment aside, we cannot but see that those who are thus forced out of their familiar habitat will benefit by the change. It is doubtful if they would come back again. Certainly their children would not. In one way the authorities might reasonably intervene. If they were to put in action the laws against overcrowding, they would limit the immigration by sheer lack of accommodation. Eviction from overcrowded dwellings would certainly mean immediate hardship to those evicted, but it would soon lessen the tension of the general situation by bringing down rents. The man who means to cram into a house twice or three times the number of persons it can decently contain, can afford to pay a high rent for it, and does so. And those who will not overcrowd suffer, for the same rent is asked from them. But the problem confessedly is a difficult one, for one desires to do justice to our own people without falling into that abyss of prejudice and persecution which marks the *Judenhetz* of the continent. All these points are touched on in Mr. Mackenzie's book, which combines a deep sympathy with the suffering poor with a welcome reasonableness and moderation of tone.

Nerves in Disorder: A Plea for Rational Treatment. By Alfred T. Schofield, M.D. (London: Hodder and Stoughton. 1903. 3s. 6d. Small 8vo. pp. xvi. and 202.)

Charcot has described a type of neurasthenic by the descriptive title of "the man with slips of paper." This type is the terror of the physician in good practice. He is full of well-arranged, well-observed symptoms, which he commits to writing, and details very exactly to his doctors and his friends. To this type of neurasthenic this book will be a godsend, a mine from which to extract fresh symptoms. It may not do much harm to the patients of this variety, because they do not want to get well, they never do get well, and they thoroughly enjoy their poor health for a lifetime. But we cannot believe this volume to be a useful one for the public at large, or for the many unfortunate people whose nervous condition is the text of the sermon. The book is a simple exposition on neurasthenia and allied conditions, giving a large space to their management and treatment. The style is simple, concise, and free from padding and "falutin'." The author's knowledge of his subject is accurate and his reading sound, and there is no question that his volume will be very helpful to physicians who have had limited experience with these difficult cases and to nurses who have to handle them. But we cannot agree with the author that it is altogether a proper guide to be widely disseminated among the general public.

